# Dysbiosis in the Gut Microbiota of Patients with Multiple Sclerosis, with a Striking Depletion of Species Belonging to *Clostridia* XIVa and IV Clusters

**DOI:** 10.1371/journal.pone.0137429

**Published:** 2015-09-14

**Authors:** Sachiko Miyake, Sangwan Kim, Wataru Suda, Kenshiro Oshima, Masakazu Nakamura, Takako Matsuoka, Norio Chihara, Atsuko Tomita, Wakiro Sato, Seok-Won Kim, Hidetoshi Morita, Masahira Hattori, Takashi Yamamura

**Affiliations:** 1 Department of Immunology, National Institute of Neuroscience, 4-1-1, Ogawa-Higashi, Kodaira, Tokyo 187–8502, Japan; 2 Department of Immunology, Juntendo University School of Medicine, 2-1-1, Hongo, Bunkyo-ku, Tokyo 113–8421, Japan; 3 Center for Omics and Bioinformatics, The Department of Computational Biology, Graduate School of Frontier Sciences, The University of Tokyo, Kashiwanoha 5-1-5, Kashiwa, Chiba 277–8561, Japan; 4 Laboratory for Integrated Bioinformatics, RIKEN Center for Integrative Medical Sciences, 1-7-22 Suehiro-cho, Tsurumi-ku, Yokohama, Kanagawa 230–0045, Japan; 5 School of Veterinary Medicine, Azabu University, 1-17-71 Fuchinobe, Chuo-ku, Sagamihara, Kanagawa 229–8501, Japan; University of Illinois at Urbana-Champaign, UNITED STATES

## Abstract

The pathogenesis of multiple sclerosis (MS), an autoimmune disease affecting the brain and spinal cord, remains poorly understood. Patients with MS typically present with recurrent episodes of neurological dysfunctions such as blindness, paresis, and sensory disturbances. Studies on experimental autoimmune encephalomyelitis (EAE) animal models have led to a number of testable hypotheses including a hypothetical role of altered gut microbiota in the development of MS. To investigate whether gut microbiota in patients with MS is altered, we compared the gut microbiota of 20 Japanese patients with relapsing-remitting (RR) MS (MS20) with that of 40 healthy Japanese subjects (HC40) and an additional 18 healthy subjects (HC18). All the HC18 subjects repeatedly provided fecal samples over the course of months (158 samples in total). Analysis of the bacterial 16S ribosomal RNA (rRNA) gene by using a high-throughput culture-independent pyrosequencing method provided evidence of a moderate dysbiosis in the structure of gut microbiota in patients with MS. Furthermore, we found 21 species that showed significant differences in relative abundance between the MS20 and HC40 samples. On comparing MS samples to the 158 longitudinal HC18 samples, the differences were found to be reproducibly significant for most of the species. These taxa comprised primarily of clostridial species belonging to *Clostridia* clusters XIVa and IV and *Bacteroidetes*. The phylogenetic tree analysis revealed that none of the clostridial species that were significantly reduced in the gut microbiota of patients with MS overlapped with other spore-forming clostridial species capable of inducing colonic regulatory T cells (Treg), which prevent autoimmunity and allergies; this suggests that many of the clostridial species associated with MS might be distinct from those broadly associated with autoimmune conditions. Correcting the dysbiosis and altered gut microbiota might deserve consideration as a potential strategy for the prevention and treatment of MS.

## Introduction

The incidence of multiple sclerosis (MS), an inflammatory disease of the central nervous system, is increasing in developed countries [1,2]. The predominance of MS-associated single nucleotide polymorphisms in genes involved in cellular immune responses and the efficacy of immune-targeted therapy in MS have indicated that it is a T cell-mediated autoimmune disease. Although >100 single nucleotide polymorphisms are linked with MS susceptibility, it is also influenced by environmental factors such as infection with Epstein-Barr virus, cigarette smoking, and lower exposure to sunlight [3,4]. However, none of these known risk factors is sufficient to account for the striking increase in the incidence of MS in Asian countries including Japan [2,5], implying that an additional environmental factor is involved. We have postulated that such an environmental risk could be related to the rapid change in the life style in Asia, particularly westernization [6].

Prior studies have conclusively shown that the gut microbiota is an essential factor that influences cellular and humoral components of the intestinal immune system [7]. More recently, the relationship between the gut microbiota and systemic immune responses, including autoimmune responses, has garnered considerable attention with regard to the pathogenesis of immune-mediated diseases. Notably, experimental approaches using experimental autoimmune encephalomyelitis (EAE), a rodent MS model, have successfully proven that alterations of the gut microbiota are a potential risk factor for developing autoimmune diseases such as MS. Supportive of this postulate, a study by Yokote *et al*. in 2008 found that alteration of the gut microbiota by oral antibiotic administration reduced the severity of conventional EAE [8]. Moreover, by using a T cell receptor transgenic mouse model of EAE, Berer *et al*. demonstrated that EAE-resistant germ-free mice develop EAE spontaneously without any manipulation with adjuvant or pertussis toxin after recolonization with indigenous bacteria or a strain of segmented filamentous bacteria [9,10]. Restoration of EAE was associated with induction of enteric Th17 cells. In addition to disease-promoting bacteria in the rodent intestine, human gut bacteria that potentially suppress local or systemic inflammation have been identified. Recent studies have shown that *Clostridia* clusters XIVa, IV, and *Bacteroides fragilis* derived from human feces have the potential to induce Foxp3^+^ regulatory T cells (Treg) and are able to suppress inflammatory conditions such as colitis and EAE [11–13].

These experiments indicate a role for the indigenous gut microbiota in the pathogenesis of autoimmune diseases, thereby raising the possibility that an altered gut microbiota is an environmental risk factor for MS. Therefore, we compared the gut microbiota of patients with MS and healthy subjects by using a high-throughput culture-independent pyrosequencing method. Bacterial 16S ribosomal RNA (rRNA) gene analysis of DNA isolated from fecal samples revealed the presence of a moderate dysbiosis in the gut microbiota of patients with MS. Moreover, we detected a significant change in the abundance of several taxa, including species belonging to *Clostridia* clusters XIVa and IV, which are known to exhibit anti-inflammatory effects [11]. Collectively, the results of the present study suggest a meaningful association between altered gut microbiota and the pathogenesis of MS and may be of relevance to the future development of novel preventive or therapeutic strategies for MS.

## Materials & Methods

### Subjects

Twenty patients with MS (MS20; aged 36.0 ± 7.2 years, 6 men and 14 women) were recruited at the National Center of Neurology and Psychiatry Hospital (S1 Table). Patients with MS that were included in the study fulfilled McDonald’s diagnostic criteria and all patients exhibited the relapsing-remitting (RR) phenotype in which inflammation mediated by autoimmune T cells and B cells plays a predominant role [14]. Patients with primary or secondary progressive MS accompanying progressive neurodegenerative pathology and other disease complications were excluded to specifically evaluate the association between the RR phenotype of patients with MS and the gut microbiota structure. The fecal samples were collected during remission phase.

In total, 50 volunteers (aged 27.2 ± 9.2 years, 23 men and 27 women) were recruited as healthy controls (HC) at Azabu University (S2 Table). None of the subjects was treated with antibiotics during collection of fecal samples. The National Center of Neurology and Psychiatry Ethics Committee, the Hospital Ethics Committee at Juntendo University Hospital, the Human Research Ethics Committee of Azabu University, and the Research Ethics Committee of the University of Tokyo approved the protocol, and written informed consent was obtained from all the subjects.

### Fecal sample collection and DNA preparation

In accordance with a previously described method [15], freshly collected fecal samples were transported at 4°C to the laboratory in a plastic bag containing a disposable oxygen-absorbing and carbon dioxide-generating agent in which anaerobes sensitive to oxygen can survive. In the laboratory, the fecal samples were suspended in phosphate-buffered saline containing 20% glycerol, immediately frozen using liquid nitrogen, and stored at -80°C until use. Bacterial DNA was isolated and purified from the fecal samples according to the previously described enzymatic lysis method [15].

Samples of 40 of the 50 healthy subjects (HC40; aged 28.5 ± 9.8 years) were used for comparison with the MS20 samples to evaluate differences in the overall microbiota structure and to identify bacterial species that differ in abundance between HC40 and MS20 samples. An additional 158 longitudinal samples were obtained from 18 healthy subjects (HC18; aged 21.9 ± 3.1 years) [15]. These healthy volunteers, including 10 additionally recruited subjects and 8 subjects from the HC40 cohort, provided fecal samples every 2 weeks (S2 Table) [15]. Using these longitudinally collected HC18 samples, bacterial species that differed significantly in abundance between HC40 and MS20 samples were further validated and the consistency of these differences in the abundance over time was examined.

### Pyrosequencing of the 16S rRNA gene V1-V2 region

The 16S rRNA gene V1-V2 region was amplified by PCR with barcoded 27Fmod (5′-agrgtttgatymtggctcag-3′) and the reverse primer 338R (5′-tgctgcctcccgtaggagt-3′) [15]. PCR was performed using 50 μl of 1× Ex Taq PCR buffer composed of 10 mM Tris-HCl (pH 8.3), 50 mM KCl, and 1.5 mM MgCl_2_ in the presence of 250 μM dNTPs, 1 U Ex Taq polymerase (Takara Bio, Kyoto, Japan), forward and reverse primers (0.2 μM), and ~20 ng of template DNA. Thermal cycling was performed in a 9700 PCR System (Life Technologies Japan, Tokyo, Japan) and the cycling conditions were as follows: initial denaturation at 96°C for 2 min, followed by 25 cycles of denaturation at 96°C for 30 s, annealing at 55°C for 45 s, and extension at 72°C for 1 min, and a final extension at 72°C. PCR amplicons were purified using AMPure XP magnetic purification beads (Beckman Coulter Inc., Brea, CA, USA) and quantified using the Quant-iT PicoGreen dsDNA Assay Kit (Life Technologies Japan). An equal amount of each PCR amplicon was mixed and subjected to sequencing with the 454 GS FLX Titanium or 454 GS JUNIOR platform (Roche Applied Science, Indianapolis, IN, USA), according to the manufacturer’s instructions.

### Construction of the full-length 16S rRNA gene database

A 16S rRNA gene database was constructed from the full-length 16S rRNA gene (FL-16S) sequences updated in the RDP, CORE, and NCBI genome databases as follows. First, high-quality FL-16S sequences were obtained by removing sequences that have <1,400 bp in length, those that contained ≥4 ambiguous bases, and those that were putatively eukaryotic from the three databases (221,537 sequences in total). These high-quality FL-16S sequences (154,850 sequences) were clustered using USEARCH5 with a 99.8% identity cut-off, resulting in 87,558 groups that consisted of identical or near-identical FL-16S sequences, representing non-redundant FL-16S sequences. The obtained 16S database was used for taxonomic assignment and quantitative mapping of pyrosequencing reads of the 16S rRNA gene V1-V2 region generated from DNA isolated from the fecal samples obtained from healthy subjects and patients with MS.

### Analysis of the 16S rRNA gene V1-V2 region

The analytical pipeline previously established for pyrosequencing reads of the 16S rRNA gene V1-V2 region (16S reads) was used [15,16]. In brief, 3,000 high-quality 16S reads, with an average quality value > 25, were randomly chosen from all filter-passed reads for each sample, and both primer sequences were trimmed. The number of operational taxonomic units (OTUs) for each sample was obtained by clustering the 3,000 16S reads with a 96% identity threshold, and this number was used to evaluate species diversity and richness. The 16S reads were then mapped to FL-16S sequences, based on a similarity search against 87,558 non-redundant FL-16S sequences by using BLAST with ≥96% identity and ≥90% coverage in sequence alignment. The FL-16S sequences mapped by 16S reads were further clustered using USEARCH5 with a 97% identity cut-off to generate clusters of FL-16S equivalent to OTUs at the species level, defined as “rclust” in this study. Taxonomic assignment of 16S reads was performed based on the 97% FL-16S clusters to which they were mapped. Unmapped 16S reads were subjected to conventional clustering by using USEARCH5 with a 96% identity cut-off to obtain the OTUs defined as “unmap_OTUs” and were assigned to a higher-level taxon (i.e., above the species level) based on a similarity search against the 16S database. The number of 16S reads that mapped to each rclust and formed each unmap_OTU was used to estimate the bacterial composition at the species, genus, and phylum levels. The 16S rRNA gene V1-V2 region sequences from healthy subjects and patients with MS that were analyzed in the present study were deposited in DDBJ/GenBank/EMBL with the accession numbers DRA000672, DRA000673, DRA000675, DRA000676, DRA000678–DRA000684, and DRA002866–DRA002874 for 20 patients with MS and DRA002875–DRA002906 for 20 healthy subjects. The accession numbers for the 16S sequences of the additional 18 healthy subjects (HC18) including the 8 subjects from the HC40 cohort were reported previously [15].

### UniFrac distance analysis

UniFrac distance analysis, a phylogenic tree-based metric, was used to measure differences in the overall bacterial gut microbiota structure [17]. Estimated OTU richness for each sample based on the Chao 1 estimator was calculated with the vegan package (v2.0–5) implemented in R (v2.15.2).

### Statistical and other analysis

All statistical analyses were conducted with R version 2.15.2. Microbial richness, evenness, and diversity were assessed using the R Vegan package. Welch's *t*-test was used for statistical analysis. *P*-values were corrected for multiple testing by using the Benjamini-Hochberg method.

## Results

### Cluster assignment of 16S pyrosequencing reads by using the FL-16S database

Using the 454 GS FLX Titanium platform, we obtained 141,549 (7,080 ± 825 reads per sample) and 303,585 (7,590 ± 616 reads per sample) high-quality 16S reads from DNA isolated from fecal samples obtained from MS20 and HC40 subjects, respectively (S3 and S4 Tables). We randomly selected 180,000 reads (3,000 reads per sample) from among the filter-passed reads to analyze species richness and diversity because clustering of excessive 16S reads with an average error rate of approximately 0.5% results in an overestimation of the species number [15].

Furthermore, we performed direct BLAST searches of 16S reads against the FL-16S database to determine taxonomic assignments; this method minimized the generation of orphan OTUs often observed in conventional clustering of error-prone 16S reads. The FL-16S database was constructed from publicly available 16S databases including the RDP, CORE, and NCBI genome databases (see Materials and Methods). We first removed sequences that measured <1,400 bp in length that may therefore not be full-length (58,823 sequences), those containing ≥4 ambiguous bases (7,377 sequences), and those that were possibly eukaryotic (487 sequences) based on sequences derived from the three databases (221,537 sequences in total) to obtain 154,850 high-quality FL-16S sequences. Then, non-redundant 16S sequences were obtained by clustering the FL-16S sequences using USEARCH5 with a 99.8% identity threshold; identical and near-identical FL-16S sequences were clustered. Finally, our own FL-16S database was constructed, consisting of 87,558 non-redundant FL-16S sequences measuring ≥1,400 bp in length and containing fewer than three unknown bases (N ≤ 3).

When the 180,000 filter-passed reads obtained from MS20 and HC40 samples were subjected to similarity searches using BLAST with ≥96% identity and ≥90% coverage in sequence alignment against the FL-16S database, 163,691 reads (109,891 from HC40 and 53,800 from MS20) were mapped to 9,816 non-redundant FL-16S clusters, and the remaining 16,309 reads (10,109 from HC40 and 6,200 from MS20) were unmapped. These data indicated that approximately 91% of the total 16S reads could be assigned to known species or strains. In addition, the proportion of unmapped reads was 8.4% for HC40 and 10.3% for MS20, respectively, suggesting a slightly higher abundance of unknown bacteria at the species level in MS20 compared to HC40.

The FL-16S sequences mapped by 16S reads were then clustered using USEARCH5 with a 97% identity threshold to generate 760 FL-16S clusters exhibiting species-level similarity. Of the clusters, 659 minor clusters consisting of 16S reads with an average relative abundance of <0.1% in both HC40 and MS20 samples were excluded from the analysis. Similarly, conventional clustering of the 16,309 unmapped reads with 96% identity generated 1,321 OTUs, of which 1,292 were not subjected to further analysis because the average relative abundance was not >0.1% in either of the groups. The remaining 29 OTUs for which the average relative abundance was ≥0.1% in either of the groups were further analyzed. Finally, 101 clusters of FL-16S sequences defined as rclust and 29 OTUs defined as unmap_OTUs were used to characterize the gut microbiota of MS20 and HC40 subjects (S1 Fig). These clusters included 163,726 reads (109,913 from HC40 and 53,813 from MS20) in total, accounting for about 91% of the total 180,000 reads used.

### Comparison of the gut microbiota between patients with MS and healthy subjects

**Fig 1:**
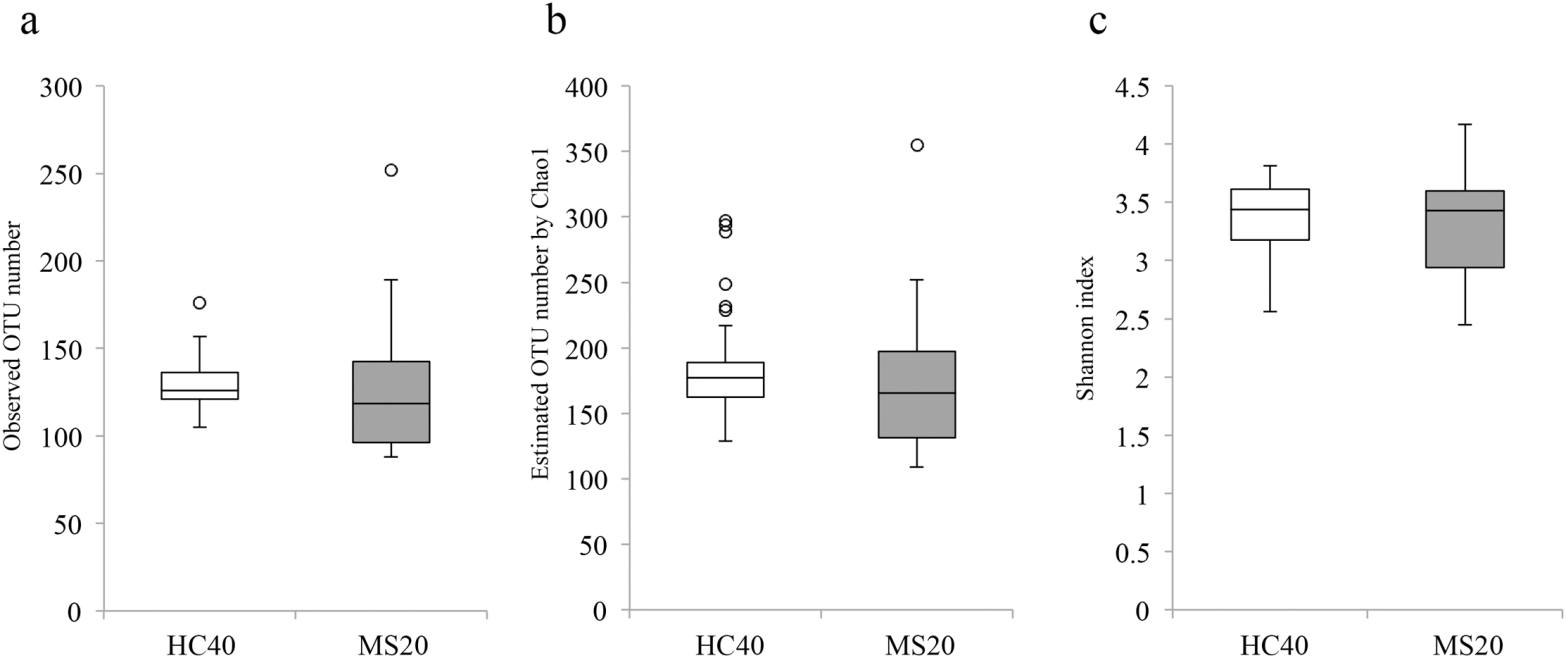
OTU/species diversity and richness in gut microbiota of HC40 and MS20 subjects. (a) Number of operational taxonomic units (OTUs) generated by clustering of 3,000 16S reads of gut microbiota samples from 40 healthy control subjects (HC40) and 20 patients with multiple sclerosis (MS20). (b) Estimated OTU numbers obtained from Chao1 extrapolation of the observed OTU numbers shown in (a). (c) Shannon index calculated from the observed OTU numbers. The vertical axes indicate the numbers of OTUs (a, b) and the Shannon index (c). Each box plot represents median, interquartile range, minimum, and maximum values.

**Fig 2:**
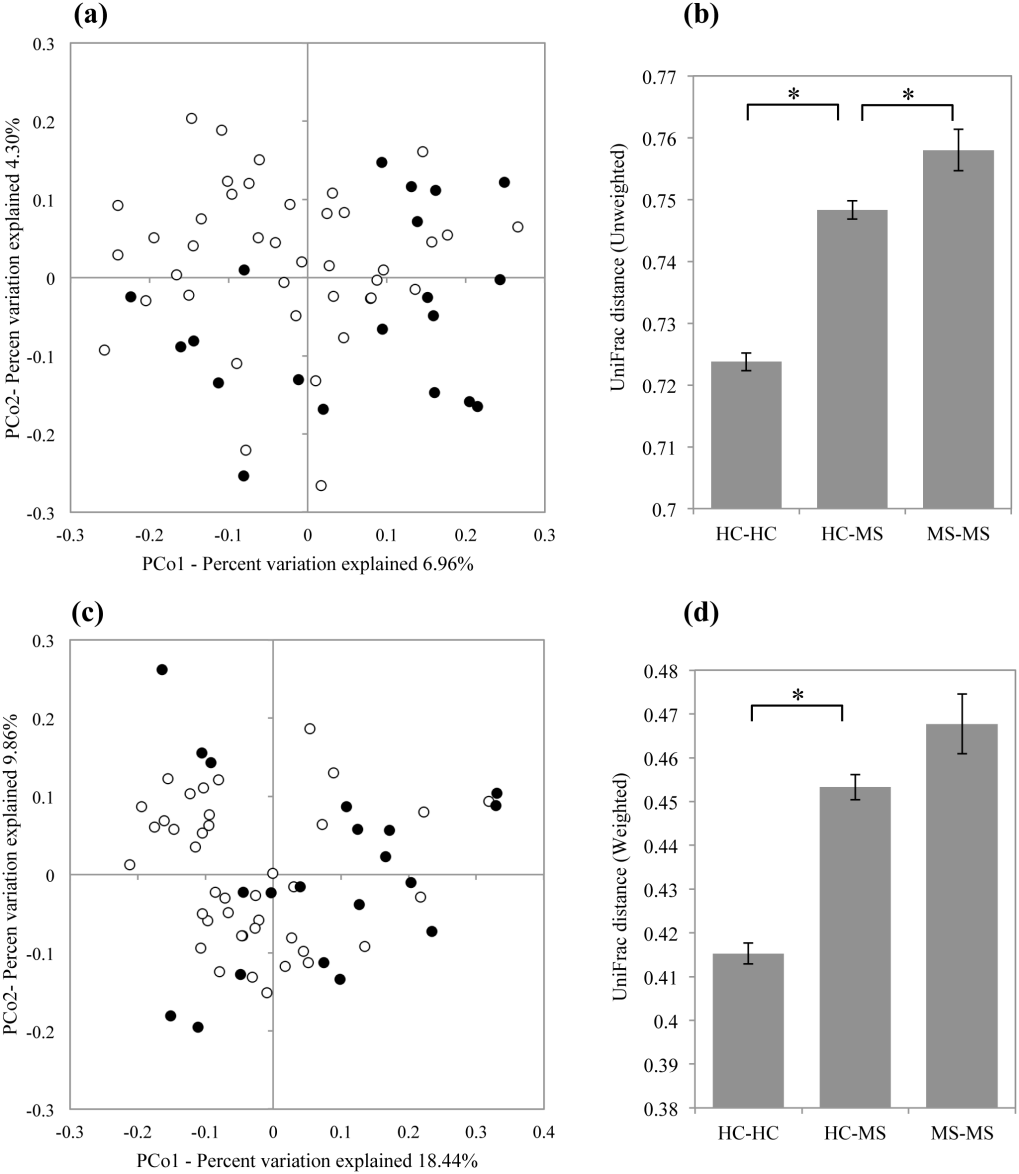
UniFrac Principal Coordinate (PCoA) and UniFrac distance analyses for HC40 and MS20 subjects. (a, c) Open and closed circles indicate individual subjects from HC40 and MS20, respectively. (a) The two components of the unweighted PCoA plot explained 6.96% and 4.30% of the variance. ANOSIM statistic, *R* = 0.239, *P* ≤ 0.0009. (b) Mean unweighted UniFrac distances for HC-HC, HC-MS, and MS-MS subjects. (c) The two components of the weighted PCoA plot explained 18.44% and 9.86% of the variance. ANOSIM statistic, *R* = 0.208, *P* ≤ 0.002. (d) Mean weighted UniFrac distances for HC-HC, HC-MS, and MS-MS subjects. (b, d) Error bars represent standard deviations of the UniFrac distances between samples. *P ≤ 0.05.

The observed mean and Chao1-estimated OTU/species number for MS20 (126.9 and 172.8, respectively) subjects were slightly lower than those for HC40 subjects (129.4 and 184.8, respectively); however, the difference in species number and richness between the groups was not statistically significant (Fig 1a and 1b). Furthermore, the Shannon index, a metric for evaluating bacterial diversity, was not significantly different between the samples obtained from HC40 (3.39 ± 0.29) and MS20 subjects (3.29 ± 0.46) (Fig 1c). We then performed UniFrac Principal Coordinate Analysis (PCoA) and UniFrac distance analysis [17]. Both unweighted and weighted UniFrac analyses showed a significant difference (*P* < 0.05) in the overall gut microbiota structure between HC40 and MS20 subjects (Fig 2a–2d). The data also revealed significantly higher inter-individual variability in the gut microbiota of MS20 subjects compared to HC40 subjects.

**Fig 3:**
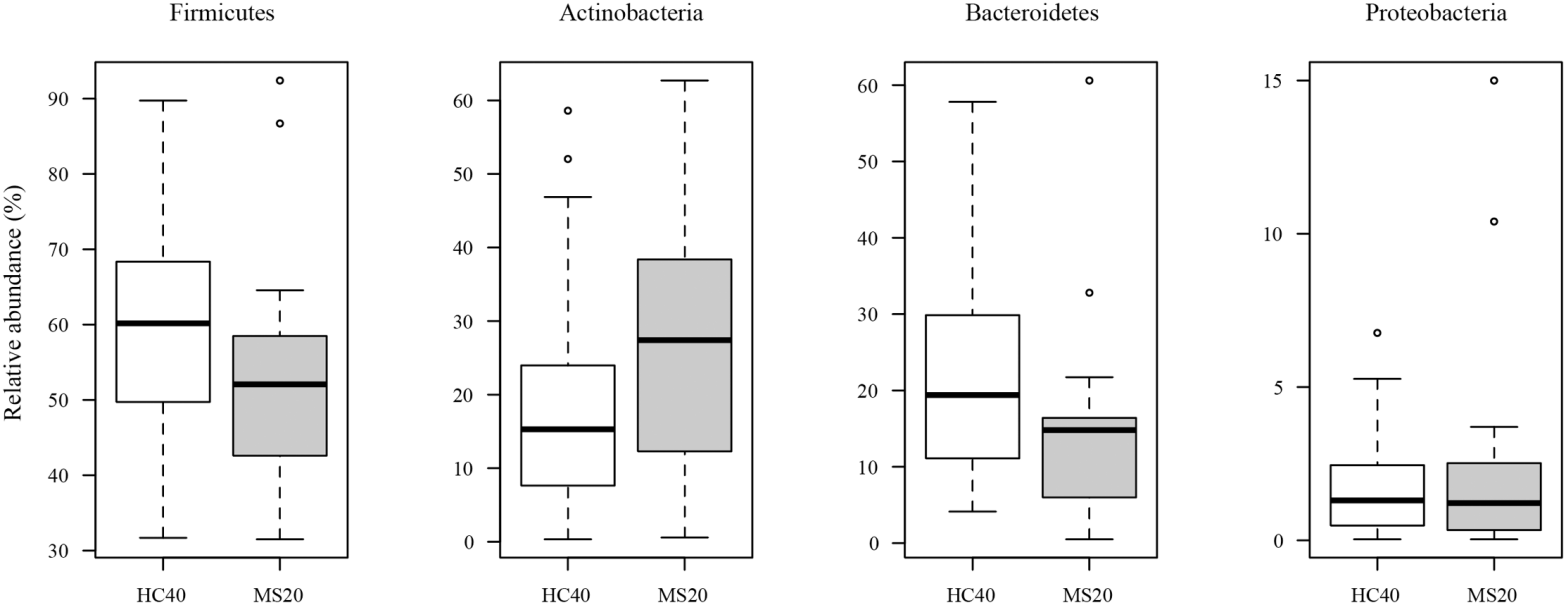
Bacterial composition at the phylum level in gut microbiota samples obtained from HC40 and MS20 subjects. For phylum-level assignment of 16S reads (16S rRNA gene V1-V2 region) mapped to the known FL-16S sequences and unmapped OTUs (see Results), 70% sequence identity threshold was applied. The vertical axis represents the relative abundance of each phylum in the microbiota of HC40 (open bar) and MS20 (grey bar) subjects. Each box plot represents median, interquartile range, minimum, and maximum values.

**Fig 4:**
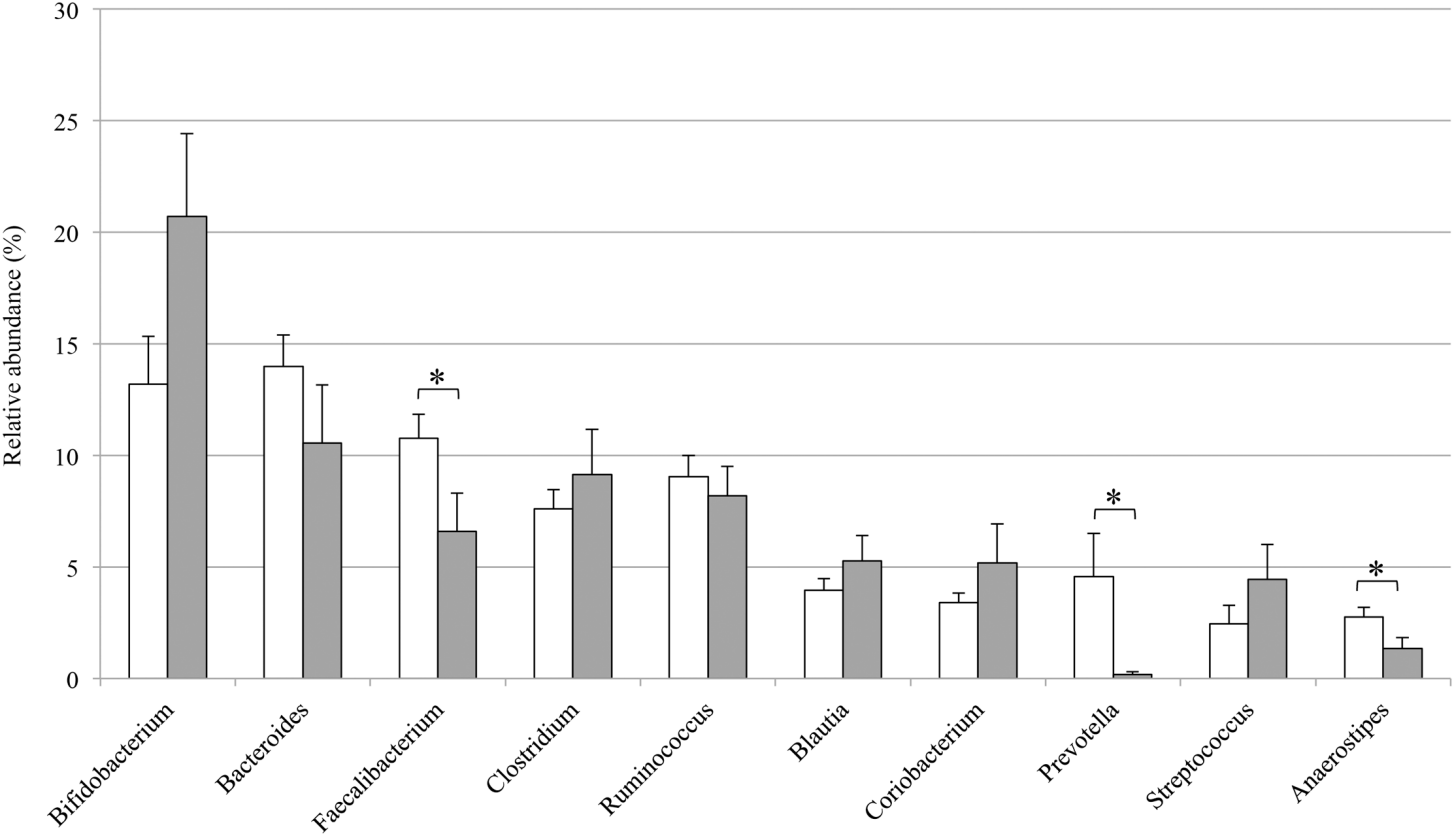
Bacterial composition at the genus level in gut microbiota samples obtained from HC40 and MS20 subjects. For genus-level assignment of 16S reads (16S rRNA gene V1-V2 region) mapped to the known FL-16S sequences and unmapped OTUs (see Results), 94% sequence identity threshold was applied. The vertical axis represents the relative abundance (%) of each genus in the microbiota of HC40 (open bar) and MS20 (grey bar) subjects. Error bars represent standard error of the mean. Asterisks indicate statistical significance determined by Welch’s *t* test (*P < 0.05).

To identify the bacterial species that exhibited significant differences in abundance between MS20 and HC40 samples, we analyzed the bacterial composition at various taxonomic levels. Taxonomic assignment at the phylum level indicated that the gut microbiota in both MS20 and HC40 subjects consisted of four major phyla, *Firmicutes*, *Bacteroidetes*, *Actinobacteria*, and *Proteobacteria*. Based on a comparative analysis, species belonging to *Actinobacteria* were more prevalent in samples obtained from MS20 subjects than in those obtained from HC40 subjects, while species belonging to *Bacteroidetes* and *Firmicutes* were less abundant in samples obtained from MS20 subjects than in those obtained from HC40 subjects. However, these differences in the representation of phyla were not statistically significant (Fig 3). A genus-level analysis revealed that, among the 10 major genera, species belonging to *Bacteroides*, *Faecalibacterium*, *Prevotella*, and *Anaerostipes* were less abundant in the gut microbiota of MS20 subjects than in that of HC40 subjects, while the *Bifidobacterium* and *Streptococcus* genera tended to be more abundant in the microbiota of MS20 subjects than in that of HC40 subjects (Fig 4).

**Fig 5:**
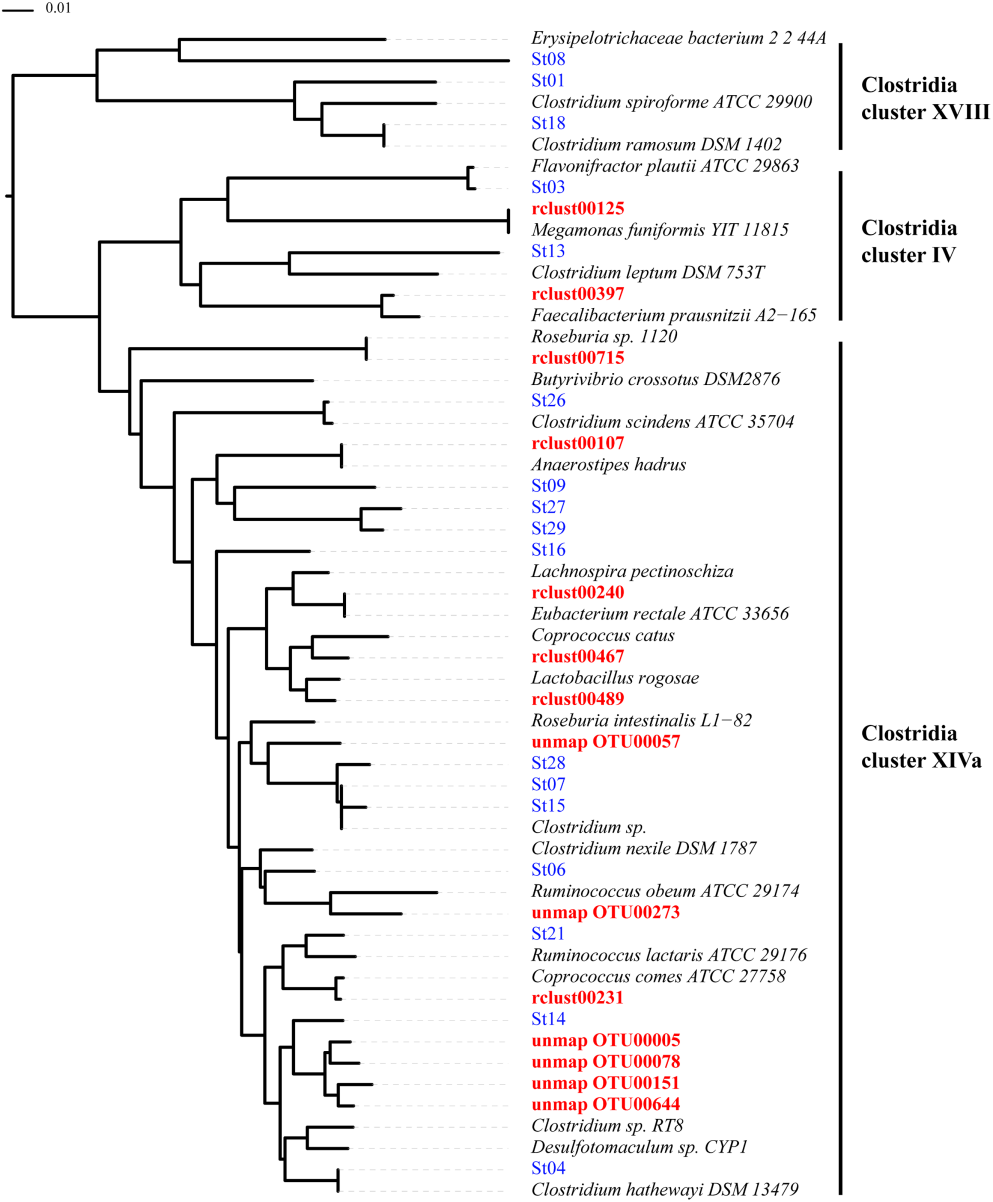
Phylogenetic tree of 14 clostridial species exhibiting significant differences among groups and several known species. The neighbor-joining method was used to construct the phylogenetic tree. Numbers at each node indicate the bootstrap support (1,000 replicates). Those written in red letters are 14 clostridial species having a significant difference in relative abundance between HC40 and MS20 samples. Treg-inducing strains are indicated by “St” and are written in blue letters [13].

We compared the relative abundance of 130 clusters equivalent to species between samples obtained from HC40 and MS20 subjects (S1 Fig). From this species-level analysis, we detected 21 species that showed significant differences (*P* < 0.05) in relative abundance between both groups (Table 1 and S2 Fig, and the detailare in S5 Table). Among them, 15 species defined as rclust exhibited ≥96% identity to known FL-16S sequences and 6 species defined as unmap_OTUs were obtained from the clustering of unmapped reads exhibiting <96% identity to known FL-16S sequences. Two species were more abundant and 19 were less abundant in the microbiota of MS20 subjects than in that of HC40 subjects. The 16S-based taxonomic assignment of the 21 species revealed that 4 species were assigned to the phylum *Bacteroidetes*, 1 was assigned to *Actinobacteria*, 1 was assigned to *Proteobacteria*, and 15 were assigned to *Firmicutes*. To determine the detailed taxonomic position of the 14 species assigned to the clostridial clade among 15 *Firmicutes* species, a phylogenetic tree was constructed based on the 16S rRNA gene V1-V2 region sequences of the 14 species and those of additional known species, including 17 clostridial strains that were shown to induce Tregs in the colon [13]. As shown in Fig 5, 12 species were located in *Clostridia* cluster XIVa and 2 were located in *Clostridia* cluster IV, all of which were less abundant in the microbiota of MS20 subjects. Interestingly, none of these 14 clostridial species was phylogenetically close to the 17 chloroform-resistant, spore-forming, Treg-inducing clostridial strains (Fig 5). The phylogenetic distinction between these two clostridial subsets was also confirmed by the fact that the sequence similarity of the 16S rRNA gene V1-V2 region in pairs between the 14 clostridial species and the 17 Treg-inducing species described by Atarashi [13] was ≤95% (S6 Table). Five other less abundant species in MS20 samples were classified into three genera: *Bacteroides* (*B*. *stercoris*, *B*. *coprocola*, and *B*. *coprophilus*), *Prevotella* (*P*. *copri*), and *Sutterella* (*S*. *wadsworthensis*).

**Table 1:** The 21 species exhibiting significant increases or decreases in abundance between HC40 and MS20 samples.

Cluster	Best hit species	Identity (%)	**Log10 (MS/HC)	*P*-value
rclust00410	Eggerthella lenta	100	0.45	0.03989
rclust00054	Streptococcus thermophiles/salivarius	100.0/99.7	0.44	0.01652
rclust00397	Faecalibacterium prausnitzii	99	-0.23	0.04167
rclust00107	Anaerostipes hadrus	100	-0.36	0.03991
rclust00240	Eubacterium rectale ATCC 33656	100	-0.43	0.02201
unmap_OTU00057	Clostridium sp.	93.8	-0.46	0.00553
rclust00231	butyrate-producing bacterium SL7/1	99.4	-0.5	0.04398
unmap_OTU00078	Clostridium sp. RT8	94.7	-0.63	0.00183
rclust00019	Bacteroides stercoris	100	-0.66	0.01038
rclust00024	Bacteroides coprocola	99.4	-0.82	0.0342
rclust00489*	Lactobacillus rogosae	96	-0.82	0.00098
	Lachnospira pectinoschiza	94.5		
rclust00715*	Roseburia sp.1120	99.4	-0.83	0.00359
	Clostridiaceae bacterium SH032	85.4		
rclust00226	Sutterella wadsworthensis 2_1_59BFAA	100	-0.86	0.02023
unmap_OTU00273	Clostridium sp. ID5	92.6	-0.92	0.04574
rclust00268	Bacteroides coprophilus	100	-1.07	0.03034
unmap_OTU00005	Clostridium sp. RT8	94.4	-1.08	0.00001
rclust00467	butyrate-producing bacterium A2-175	99.7	-1.11	0.00021
unmap_OTU00644	Desulfotomaculum sp. CYP1	92.9	-1.11	0.00161
unmap_OTU00151	Clostridium sp. RT8	92.6	-1.15	0.00237
rclust00255	Prevotella copri DSM 18205	99.1	-1.43	0.029
rclust00125	Megamonas funiformis YIT 11815	99.1	-1.77	0.00648

*Species with the secondly highest similarity are also indicated for rclust00489 and rclust00715 (see text).

**Read number = 1 was assumed for ND (not detected) for calculation of MS/HC.

Two species exhibiting a significant increase in abundance in samples obtained from MS20 subjects were identified and assigned to *Streptococcus thermophilus* and *Eggerthella lenta*, according to their highest sequence similarity to known species. In this taxonomic assignment, rclust00054, which was assigned to *S*. *thermophilus* with 100% identity, also had 99.7% identity with *S*. *salivarius*, suggesting that rclust00054 might consist of these two species that are indistinguishable by the analysis based on the 16S sequence (Table 1 and S5 Table). The species closest to rclust00231 and rclust00467 were the genus-undefined butyrate-producing bacteria with >99% identity; however, these two species also shared 97.9% and 95.2% identity with *Coprococcus comes* ATCC 27758 (accession number: NZ_ABVR00000000) and *Coprococcus catus* (accession number: S001014091), respectively, indicating that these two species can be assigned to the genus *Coprococcus* (Table 1 and S5 Table). A cluster rclust00715 shared the highest similarity with *Roseburia* sp. 1120 (accession number: S003610183; 99.4% identity). However, the 16S sequence similarity analysis revealed that *Roseburia* sp. 1120 was phylogenetically distinct from other known *Roseburia* species such as *R*. *faecis*, *R*. *intestinalis*, and *R*. *hominis* (S7 Table), indicating that *Roseburia* sp. 1120 may not be a member of the genus *Roseburia*. Since the species sharing the second-highest similarity with rclust00715 was *Clostridiaceae* bacterium SH032 (accession number: S000994782; 85.4% identity), rclust00715 can be assigned to the yet-undefined genus in *Clostridia* cluster XIVa (Table 1 and Fig 5). In addition, rclust00489 shared the highest similarity with *Lactobacillus rogosae* (accession number: S001873784; 96.0% identity). However, the 16S sequence similarity analysis revealed that *L*. *rogosae* shared as low as 75~80% sequence similarity with the other *Lactobacillus* species (S8 Table), indicating that *L*. *rogosae* has a phylogeny distinct from that of other *Lactobacillus* species and may therefore not belong to the genus *Lactobacillus*. This is in agreement with the recent description [18], and the present analysis showed that rclust00489 shared the highest similarity of 94.5% with *Lachnospira pectinoschiza*, located in *Clostridia* cluster XIVa (Table 1, Fig 5). As expected, all of the 6 unmap_OTUs exhibiting a significant difference in relative abundance between the HC40 and MS 20 samples could not be assigned to known species at the species and genus levels; however, the phylogenetic tree suggested that all of them belong to *Clostridia* cluster XIVa (Fig 5).

**Fig 6:**
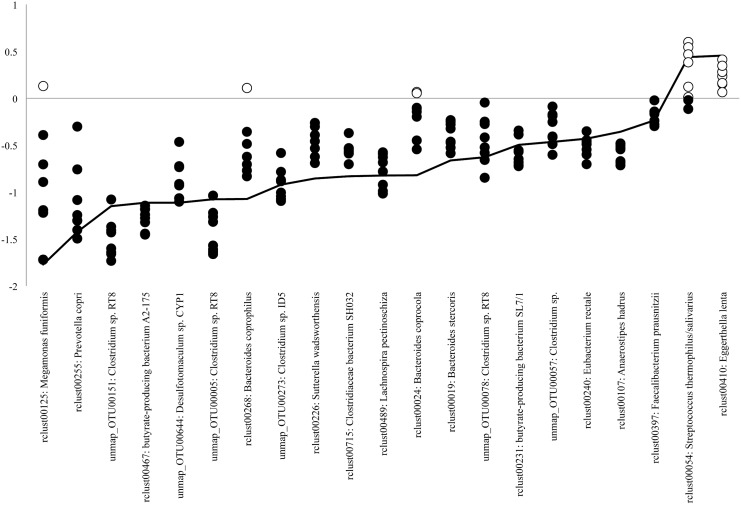
Fold-change in the abundance of 21 species using longitudinal HC18 samples. The vertical axis indicates the log value of fold-change in the abundance of 22 species for the comparison between MS20 and HC18 (nine longitudinal samples per individual) samples. Open and closed circles indicate log values of fold-change > 0 (increased in MS) and < 0 (decreased in MS), respectively. The average fold-change in the abundance of each species for the comparison between MS20 and HC40 samples was connected by a line.

To validate the significant differences in the abundance of the 21 species identified in the gut microbiota of patients with MS, we further analyzed their longitudinal fold-changes in relative abundance between MS20 and HC18 subjects, whose fecal samples were collected at 9 different time points at 2-week intervals, as previously described [15]. The results are shown in Fig 6 and indicate that the log-transformed fold-change in relative abundance for the two species that were more abundant in MS20 samples was >0 for most longitudinal HC18 samples. Similarly, the log-transformed fold-change in relative abundance for 19 less abundant species in MS20 samples was <0 for most longitudinal HC18 samples. Thus, the 21 species exhibited differences between MS20 samples and most of the longitudinal HC18 samples similar to those observed for the comparison with HC40 samples (Fig 6). These data confirmed the results obtained from the comparison between MS20 and HC40 samples.

## Discussion

The gut microbiota of Japanese patients with RR MS showed high similarity in terms of species richness with healthy controls (Fig 1). This feature is in contrast to that observed in the gut microbiota of patients with inflammatory bowel disease (IBD), which is characterized by significantly lower species richness than that observed in healthy controls [19,20]. However, UniFrac analysis revealed a significant difference (*P* < 0.05) in the overall gut microbiota structure between patients with MS and healthy controls (Fig 2). The gut microbiota of patients with MS showed higher inter-individual variability than did that of healthy controls, while patients with IBD shared high structural similarity in gut microbiota among them [21–23]. Taken together, these results suggest that the gut microbiota in patients with RR MS is characterized by moderate dysbiosis, highlighting a distinction in the composition of the gut microbiota between MS and IBD.

We identified 21 species that exhibited significant changes in relative abundance between patients (MS20) and controls (HC40), which were further validated in longitudinal samples of another healthy group (HC18). Therefore, the observed differences in abundance of the 21 species were not temporary but rather constant over time in the gut microbiota of patients with MS, suggesting that alterations in the proportion of these species would characterize the gut microbiota in Japanese patients with MS.

Among the 21 species, a depletion of 19 species was striking in MS samples, and fourteen of them belonged to *Clostridia* clusters XIVa and IV (Fig 5), in which the proportions of *Faecalibacterium prausnitzii* and *Eubacterium rectale* were reduced in fecal and mucosa-associated microbiota in patients with IBD and were associated with a higher risk of postoperative recurrence of ileal Crohn’s disease [24–26]. *Clostridia* clusters XIVa and IV are formed by highly diverse bacterial species, many of which are characterized by the ability to produce short chain fatty acids [11, 13, 24]. In particular, butyrate is implicated in colonic epithelium homeostasis, stimulation of anti-inflammatory responses for IBD [25, 26], and the induction of colonic Tregs [27–29]. Accordingly, it is conceivable that a depletion of a large subset of clostridial butyrate producers is associated with MS pathogenesis. In addition, a lack of overlap between the 14 clostridial species and the 17 Treg-inducing clostridial strains described by Atarashi, many of which are significantly reduced in samples obtained from patients with IBD [13], suggests that species from *Clostridia* clusters XIVa and IV that are associated with MS might be distinct from those associated with IBD.

The present study also found a reduction in the proportion of several *Bacteroides* including *B*. *stercoris*, *B*. *coprocola*, and *B*. *coprophilus* in the gut microbiota of patients with MS. Similarly, reduced abundance of several *Bacteroides* species was observed in fecal and mucosa-associated microbiota in patients with IBD [21, 30]. In contrast, positive correlations between *Bacteroides* and systemic inflammations were also demonstrated [31, 32]. Our data also suggested a negative correlation of *Prevotella copri* with the pathogenesis of MS, while the increased abundance of this bacterium was associated with the pathogenesis of rheumatoid arthritis, a prevalent systemic autoimmune disease [33]. The significant decrease in *Sutterella wadsworthensis* in MS is unlikely linked to IBD [34], but it was less frequently detected in ileal and cecal biopsy samples from children with gastrointestinal dysfunction than in those from children with both autism and gastrointestinal dysfunction [35]. As mentioned above, the structural and species-level alterations in the gut microbiota associated with MS in Japanese patients were not always similar to those observed in the gut microbiota of patients with other inflammatory diseases. This discrepancy may be simply due to the difference in microbe-associated immune states between MS and other diseases. Alternatively, the observed alterations might be specific for Japanese patients, similar to the geographical or population-level variations suggested for the gut microbiota of patients with Type 2 diabetes and IBD [36, 37]. Even though available information is too limited at present, we do not exclude a possibility that gut microbiota may influence on the neurodegenerative processes in MS. Further experiments using gnotobiote mice colonized by these species may help to understand whether the alterations in specific species in MS are primary or secondary to MS pathogenesis.

Farrokhi *et al*. recently reported a significantly lower level of the lipodipeptide Lipid 654, which is produced by a variety of intestinal *Bacteroidetes* species, in the serum of patients with MS than in that of healthy controls and suggested this lipid to be a useful biomarker to evaluate MS activity [38, 39]. This reduced level of Lipid 654 may be linked to the reduced relative abundance of species belonging to the *Bacteroidetes* phylum including the genera *Bacteroides* and *Prevotella* in patients with MS (Figs 3 and 4).

We did not find any significant correlation between the differences in microbial species and additional factors such as age, gender, disease duration, relapse frequency, medical treatment, and distribution of disease sites. Further studies using an increased number of MS patients will be necessary to exactly elucidate the contribution of these factors.

This may be the first report demonstrating dysbiosis of the gut microbiota in patients with MS. Our findings will facilitate the development of a novel preventive and therapeutic strategy for MS by controlling intestinal microbiota.

## Supporting Information

S1 Fig Workflow for the mapping analysis of 16S reads to the database of full-length 16S gene sequences. (TIFF)

S2 Fig Fold-change in the relative abundance of 21 species showing significant differences between HC40 and MS20 samples. Sequences of species that are the most similar to the representative 16S V1-V2 sequences of the 21 species are indicated in parentheses. Horizontal bars indicate the log-transformed fold-changes in the relative abundance of the 21 species between HC40 and MS20 samples.
(TIF)

S1 Table Clinical data of MS patients examined in this study. (XLSX)

S2 Table Samples obtained from healthy subjects for analysis in this study. (XLSX)

S3 Table Statistical summary of 16S sequencing of 20 MS patients. (XLSX)

S4 Table Statistical summary of 16S sequencing of 40 healthy subjects. (XLSX)

S5 Table Details of the 21 species having significant increase or decrease between HC40 and MS20. (XLSX)

S6 Table Similarity between the 16S V1-V2 sequences of the 14 clostridial species identified in the microbiota of patients with MS and 17 clostridial species reported by Atarashi. (XLSX)

S7 Table Similarity between the 16S V1-V2 sequences of *Roseburia* sp. 1120 and several known *Roseburia* species. (XLSX)

S8 Table Similarity between the 16S V1-V2 sequences of *Lactobacillus rogosae* and several known *Lactobacillus* species. (XLSX)

## References

[1] Bach JF. The effect of infections on susceptibility to autoimmune and allergic diseases. N Engl J Med. 2002;347: 911–920. 12239261 10.1056/NEJMra020100

[2] Osoegawa M, Kira J, Fukazawa T, Fujihara K, Kikuchi S, Matsui M, et al Temporal changes and geographical differences in multiple sclerosis phenotypes in Japanese: nationwide survey results over 30 years. Mult Scler. 2009;15: 159–173. 10.1177/1352458508098372 18987106

[3] International Multiple Sclerosis Genetics Consortium; Wellcome Trust Case Control Consortium 2, Sawcer S, Hellenthal G, Pirinen M, et al Genetic risk and a primary role for cell-mediated immune mechanisms in multiple sclerosis. Nature. 2011;476: 214–219. 10.1038/nature10251 21833088 PMC3182531

[4] Ascherio A, Munger KL. Environmental risk factors for multiple sclerosis. Part II: Noninfectious factors. Ann Neurol. 2007;61: 504–513. 17492755 10.1002/ana.21141

[5] Houzen H, Niino M, Hirotani M, Fukazawa T, Kikuchi S, Tanaka K, et al Increased prevalence, incidence, and female predominance of multiple sclerosis in northern Japan. J Neurol Sci. 2012;323: 117–122. 22995683 10.1016/j.jns.2012.08.032

[6] Yamamura T, Miyake S. Diet, gut flora, and multiple sclerosis: current research and future perspectives In: Yamamura T, Gran B, editors. Multiple sclerosis immunology. New York: Springer; 2012 pp. 115–126.

[7] Belkaid Y, Hand TW. Role of the microbiota in immunity and inflammation. Cell. 2014;157:121–141 10.1016/j.cell.2014.03.011 24679531 PMC4056765

[8] Yokote H, Miyake S, Croxford JL, Oki S, Mizusawa H, Yamamura T. NKT cell-dependent amelioration of a mouse model of multiple sclerosis by altering gut flora. Am J Pathol. 2008;173: 1714–1723. 10.2353/ajpath.2008.080622 18974295 PMC2626383

[9] Berer K, Mues M, Koutrolos M, Rasbi ZA, Boziki M, Johner C, et al Commensal microbiota and myelin autoantigen cooperate to trigger autoimmune demyelination. Nature. 2011;479: 538–541. 10.1038/nature10554 22031325

[10] Lee YK, Menezes JS, Umesaki Y, Mazmanian SK. Proinflammatory T-cell responses to gut microbiota promote experimental autoimmune encephalomyelitis. Proc Natl Acad Sci USA. 2011;108: 4612–4622. 10.1073/pnas.1000082107 PMC3063590 20660719

[11] Atarashi K, Tanoue T, Shima T, Imaoka A, Kuwahara T, Momose Y, et al Induction of colonic regulatory T cells by indigenous *Clostridium* species. Science. 2011;331:337–341. 10.1126/science.1198469 21205640 PMC3969237

[12] Ochoa-Repáraz J, Mielcarz DW, Ditrio LE, Burroughs AR, Begum-Haque S, Dasgupta S, et al Central nervous system demyelinating disease protection by the human commensal *Bacteroides fragilis* depends on polysaccharide A expression. J Immunol. 2010;185: 4101–4108. 10.4049/jimmunol.1001443 20817872

[13] Atarashi K, Tanoue T, Oshima K, Suda W, Nagano Y, Nishikawa H, et al Treg induction by a rationally selected mixture of *Clostridia* strains from the human microbiota. Nature. 2013;500: 232–236. 10.1038/nature12331 23842501

[14] McDonald WI, Compston A, Edan G, Goodkin D, Hartung HP, Lublin FD, et al Recommended diagnostic criteria for multiple sclerosis: guidelines from the International Panel on the diagnosis of multiple sclerosis. Ann Neurol. 2011;50: 121–127. 10.1002/ana.1032 11456302

[15] Kim SW, Suda W, Kim S, Oshima K, Fukuda S, Ohno H, et al Robustness of gut microbiota of healthy adults in response to probiotic intervention revealed by high-throughput pyrosequencing. DNA Res. 2013;20: 241–53. 10.1093/dnares/dst006 23571675 PMC3686430

[16] Said HS, Suda W, Nakagome S, Chinen H, Oshima K, Kim S, et al Dysbiosis of salivary microbiota in inflammatory bowel disease and its association with oral immunologic biomarkers. DNA Res. 2014;21: 15–25. 10.1093/dnares/dst037 24013298 PMC3925391

[17] Lozupone C, Lladser ME, Knight D, Stombaugh J, Knight R. UniFrac: an effective distance metric for microbial community comparison. ISME J. 2011;5: 169–172. 10.1038/ismej.2010.133 20827291 PMC3105689

[18] Tindall BJ. The status of the name Lactobacillus rogosae Holdeman and Moore 1974. Opinion 88. Judicial Commission of the International Committee on Systematics of Prokaryotes. Int J Syst Evol Microbiol. 2014; 64: 3578–3579. 10.1099/ijs.0.069146-0 25288658

[19] Ott SJ, Musfeldt M, Wenderoth DF, Hampe J, Brant O, Folsch UR, et al Reduction in diversity of the colonic mucosa associated bacterial microflora in patients with active inflammatory bowel disease. Gut. 2004;53: 685–693. 15082587 10.1136/gut.2003.025403 PMC1774050

[20] Manichanh C, Rigottier-Gois L, Boonnaud E, Gloux K, Pelletier E, Frangeul L, et al Reduced diversity of fecal microbiota in Crohn’s disease revealed by a metagenomic approach. Gut. 2006;55: 205–211. 16188921 10.1136/gut.2005.073817 PMC1856500

[21] Frank DN, St Amand AL, Feldman RA, Boedeker EC, Harpaz N, Pace NR. Molecular-phylogenetic characterization of microbial community imbalances in human inflammatory bowel diseases. Proc Natl Acad Sci USA. 2007;104: 13780–13785. 17699621 10.1073/pnas.0706625104 PMC1959459

[22] Peterson DA, Frank DN, Pace NR, Gordon JI. Metagenomic approaches for defining the pathogenesis of inflammatory bowel diseases. Cell Host Microbe. 2008;3: 417–427. 10.1016/j.chom.2008.05.001 18541218 PMC2872787

[23] Qin J, Li R, Raes J, Arumugam M, Burgdorf KS, Manichanh C, et al A human gut microbial gene catalogue established by metagenomic sequencing. Nature. 2010;464: 59–65. 10.1038/nature08821 20203603 PMC3779803

[24] Sokol H, Pigneur B, Watterlot L, Lakhdari O, Bermúdez-Humarán LG, Gratadoux JJ, et al *Faecalibacterim prausnitzii* is an anti-inflammatory commensal bacterium identified by gut microbiota analysis of Crohn disease patients. Proc Natl Acad Sci USA. 2008;105: 16731–16736. 10.1073/pnas.0804812105 18936492 PMC2575488

[25] Mondot S, Kang S, Furet JP, Aguirre de Carcer D, McSweeney C, Morrison M, et al Highlighting new phylogenetic specificities of Crohn’s disease microbiota. Inflamm Bowel Dis. 2011;17: 185–192. 20722058 10.1002/ibd.21436

[26] Joossens M, Huys G, Cnockaert M, de Preter V, Verbeke K, Rutgeerts P, et al Dysbiosis of the faecal microbiota in patients with Crohn’s disease and their unaffected relatives. Gut. 2011;60: 631–637. 10.1136/gut.2010.223263 21209126

[27] Furusawa Y, Obata Y, Fukuda S, Endo T, Nakato G, Takahashi D, et al Commensal microbe-derived butyrate induces the differentiation of the colonic regulatory T cells. Nature. 2013;504: 456–460. 24226770 10.1038/nature12721

[28] Smith PM, Howitt MR, Panikov N, Michaud M, Gallini CA, Bohlooly YM, et al The microbial metabolites, short-chain fatty acids, regulate colonic Treg cell homeostasis. Science. 2013;341: 569–573. 10.1126/science.1241165 23828891 PMC3807819

[29] Arpaia N, Campbell C, Fan X, Dikiy S, van der Veeken J, deRoos P, et al Metabolites produced by commensal bacteria promote peripheral regulatory T-cell generation. Nature. 2013;504: 451–455. 10.1038/nature12726 24226773 PMC3869884

[30] Zitomersky NL, Atkinson BJ, Franklin SW, Mitchell PD, Snapper SB, Comstock LE, et al Characterization of adherent bacteroidales from intestinal biopsies of children and young adults with inflammatory bowel disease. PLoS One. 2013;8: e63686 10.1371/journal.pone.0063686 23776434 PMC3679120

[31] Claesson MJ, Jeffery IB, Conde S, Power SE, O’Connor EM, Cusack S, et al Gut microbiota composition correlates with diet and health in the elderly. Nature. 2012;488: 178–184. 10.1038/nature11319 22797518

[32] Le Chatelier E, Nielsen T, Qin J, Prifti E, Hildebrand F, Falony G, et al Richness of human gut microbiome correlates with metabolic markers. Nature. 2013;500: 541–546. 10.1038/nature12506 23985870

[33] Scher JU, Sczesnak A, Longman RS, Segata N, Ubeda C, Bielski C, et al Expansion of intestinal Prevotella copri correlates with enhance susceptibility to arthritis. Elife. 2013; 5: e01202. 10.7554/eLife.01202 PMC3816614 24192039

[34] Mukhopadhya I, Hansen R, Nicholl CE, Alhaidan YA, Thomson JM, Berry SH, et al A comprehensive evaluation of colonic mucosal isolates of Sutterella wadsworthensis from inflammatory bowel disease. PLoS One. 2011;6: e27076 10.1371/journal.pone.0027076 22073125 PMC3205041

[35] Williams BL, Hornig M, Parekh T, Lipkin WI. Application of novel PCR-based methods for detection, quantitation, and phylogenetic characterization of Sutterella species in intestinal biopsy samples from children with autism and gastrointestinal disturbances. MBio. 2012;3: pii: e00261–11. 10.1128/mBio.00261-11 22233678 PMC3252763

[36] Karlsson FH, Tremaroli V, Nookaew I, Bergström G, Carl Johan Behre CJ, Fagerberg B, et al Gut metagenome in European women with normal, impaired and diabetic glucose control. Nature. 2013;498: 99–103. 10.1038/nature12198 23719380

[37] Rehman A, Rausch P, Wang J, Skieceviciene J, Kiudelis G, Bhagalia K, et al Geographical patterns of the standing and active human gut microbiome in health and IBD. Gut. 2015 pii: gutjnl-2014-308341. 10.1136/gutjnl-2014-308341 25567118

[38] Farrokhi V, Nemati R, Nichols FC, Yao X, Anstadt E, Fujiwara M, et al Bacterial lipodipeptide, Lipid 654, is a microbiome-associated biomarker for multiple sclerosis. Clin Transl Immunol. 2013; 2:e8. 10.1038/cti.2013.11 PMC4232052 25505950

[39] Clark RB, Cervantes JL, Maciejewski MW, Farrokhi V, Nemati R, Yao X, et al Serine lipids of *Porphyromonas gingivalis* are human and mouse Toll-like receptor 2 ligands. Infect Immun. 2013;81: 3479–3489. 10.1128/IAI.00803-13 23836823 PMC3754206

