# Supplementary figures and images for: Dysbiosis in the Gut Microbiota of Patients with Multiple Sclerosis, with a Striking Depletion of Species Belonging to Clostridia XIVa and IV Clusters
Source: PLoS One. 2015 Sep 14;10(9):e0137429. doi: 10.1371/journal.pone.0137429 (PMC4569432; doi:10.1371/journal.pone.0137429)

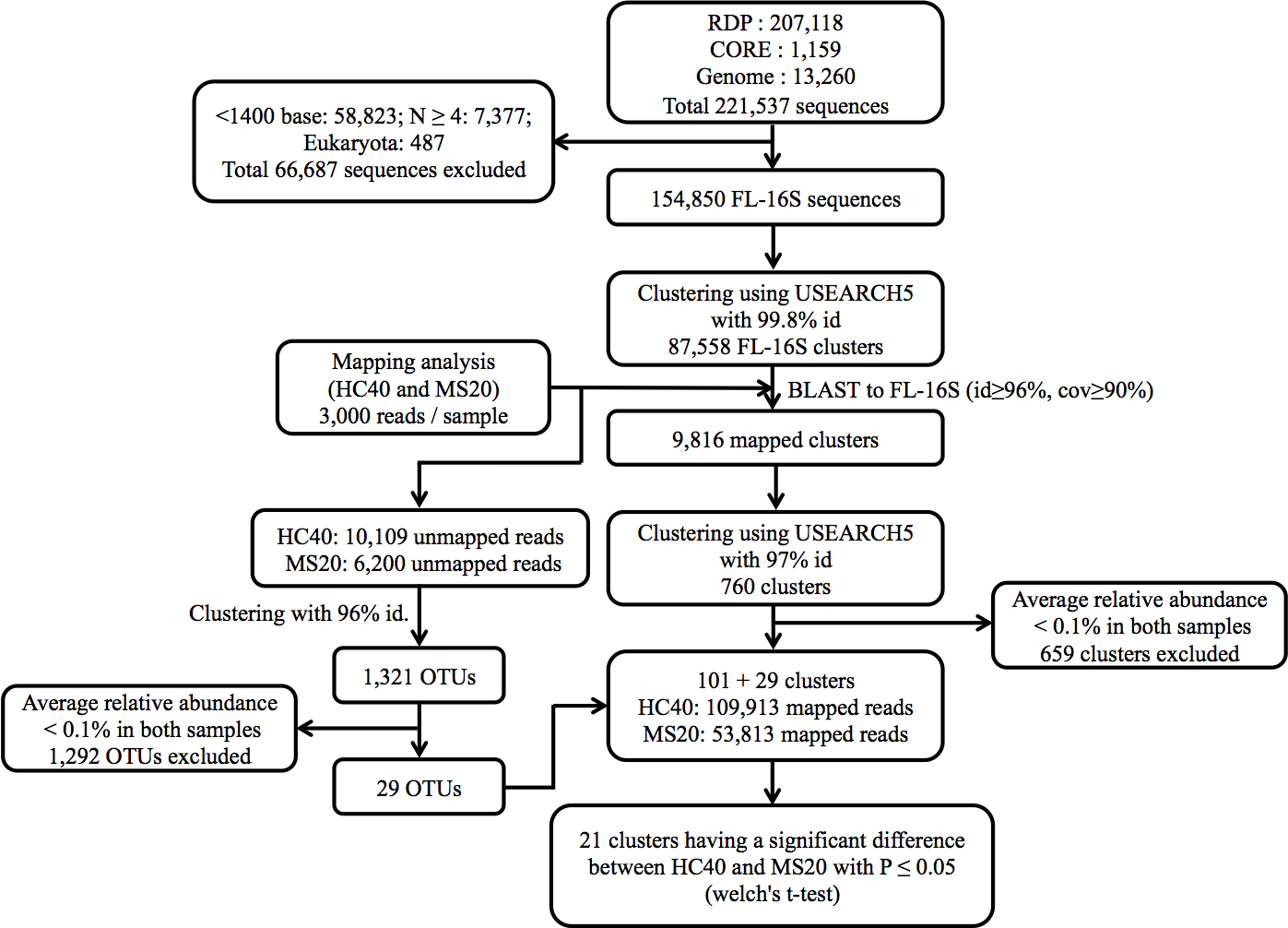

Supplement: S1 Fig — (TIFF) [file pone.0137429.s001.tiff]

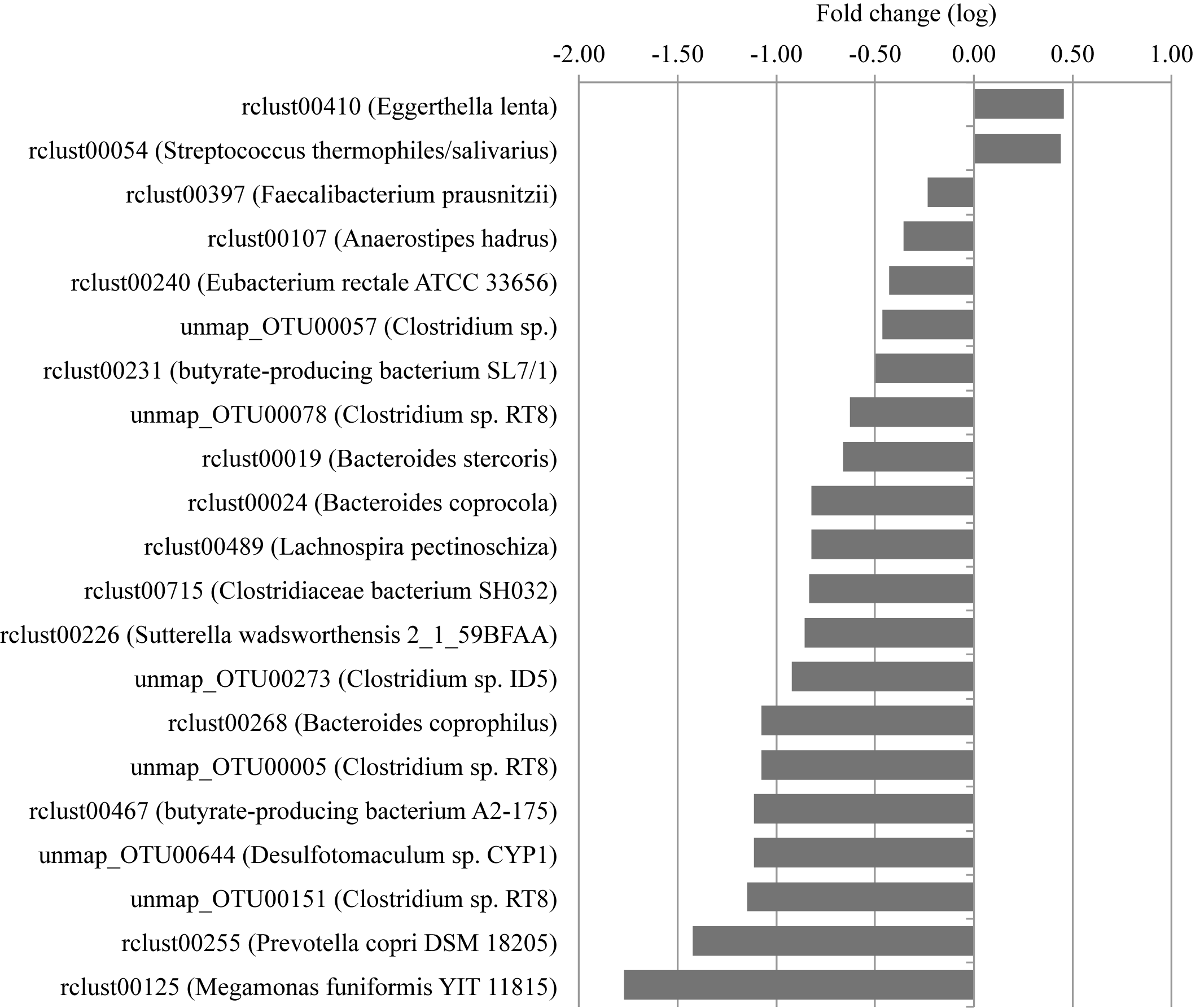

Supplement: S2 Fig — Sequences of species that are the most similar to the representative 16S V1-V2 sequences of the 21 species are indicated in parentheses. Horizontal bars indicate the log-transformed fold-changes in the relative abundance of the 21 species between HC40 and MS20 samples. (TIF) [file pone.0137429.s002.tif]
